# Single-cell transcriptome analysis reveals secretin as a hallmark of human enteroendocrine cell maturation

**DOI:** 10.1038/s41598-024-63699-0

**Published:** 2024-06-12

**Authors:** Franc Hysenaj, Michael Lauber, Andrea Bast-Habersbrunner, Markus List, Martin Klingenspor

**Affiliations:** 1 Chair of Experimental Bioinformatics, School of Life Sciences, Technical University of Munich, 85354 Freising, Germany; 2 Chair of Molecular Nutritional Medicine, School of Life Sciences, Technical University of Munich, 85354 Freising, Germany; 3 Data Science in Systems Biology, TUM School of Life Sciences, Technical University of Munich, 85354 Freising, Germany; 4 Munich Data Science Institute (MDSI), Technical University of Munich, 85748 Garching, Germany

**Keywords:** Biomarkers, Endocrinology, Gastroenterology, Gene expression analysis

## Abstract

The traditional nomenclature of enteroendocrine cells (EECs), established in 1977, applied the “one cell - one hormone” dogma, which distinguishes subpopulations based on the secretion of a specific hormone. These hormone-specific subpopulations included S cells for secretin (SCT), K cells for glucose-dependent insulinotropic polypeptide (GIP), N cells producing neurotensin (NTS), I cells producing cholecystokinin (CCK), D cells producing somatostatin (SST), and others. In the past 15 years, reinvestigations into murine and human organoid-derived EECs, however, strongly questioned this dogma and established that certain EECs coexpress multiple hormones. Using the Gut Cell Atlas, the largest available single-cell transcriptome dataset of human intestinal cells, this study consolidates that the original dogma is outdated not only for murine and human organoid-derived EECs, but also for primary human EECs, showing that the expression of certain hormones is not restricted to their designated cell type. Moreover, specific analyses into SCT-expressing cells reject the presence of any cell population that exhibits significantly elevated secretin expression compared to other cell populations, previously referred to as S cells. Instead, this investigation indicates that secretin production is realized jointly by other enteroendocrine subpopulations, validating corresponding observations in murine EECs also for human EECs. Furthermore, our findings corroborate that *SCT* expression peaks in mature EECs, in contrast, progenitor EECs exhibit markedly lower expression levels, supporting the hypothesis that *SCT* expression is a hallmark of EEC maturation.

## Author summary

It has been 120 years since the discovery of the first peptide hormone, secretin (SCT). SCT has emerged as a multifunctional regulator, not only of gastric acid and pancreatic bicarbonate secretion, but also of meal-associated thermogenesis and energy intake. However, our understanding of its expression and secretion remains limited. SCT is one of the enteroendocrine hormones, which together regulate the digestion of food and the absorption of nutrients in the digestive system. Over the past 15 years, research in this field has challenged the traditional “one cell - one hormone” classification of enteroendocrine cells (EECs). This classification assigns each type of EEC to secrete only one hormone^1^. Investigations into human EEC hormone profiles, however, are scarce and demand for the application of state of the art analysis techniques. Single-cell RNA sequencing (scRNA-seq), a cutting-edge sequencing technology, provides a high-resolution analysis of gene expression at the individual cell level, enabling the study of cellular diversity and heterogeneity within complex biological systems. In the present study, the gut cell atlas (GCA), the largest single-cell dataset of human intestinal cells currently available, was analyzed and visualized using a variety of tools. The aim was to use the transcriptional information to investigate human EEC subclassification and hormone expression landscape and to clarify the origin of SCT expression. This study showed that almost all human EECs produce multiple enteroendocrine hormones, consolidating the “one EEC-multiple hormones” principle that has previously been observed in murine and human organoid-derived EECs. The study also calls into question the existence of so-called S cells as cells that can be distinguished from other EECs solely by their high secretion of SCT. Instead, the present analysis suggests that SCT is not produced by a single cell type, but jointly by several other enteroendocrine subpopulations. In the light of emerging pharmacotherapies based on peptides of the glucagon gene family for diabetes and obesity, a better understanding of EEC heterogeneity and their hormone expression patterns could lead to the development of more targeted and effective treatments to fight the growing epidemic of obesity and diabetes, which the World Health Organization (WHO) has described as one of the most serious public health challenges of the 21st century.

## Introduction

The global prevalence of obesity has increased drastically in the past 30 years due to economic growth, industrialization, and passive lifestyles. The World Health Organization (WHO) estimates that approximately one in six adults is obese^2^. Obesity is associated with a number of chronic diseases, disabilities, and mental health problems, and carries significant economic and psychosocial costs. Treatment approaches for obesity include lifestyle modification, surgery, and pharmacotherapy, with lifestyle modification being the most common approach^3^. In this context, enteroendocrine cells (EECs), which play a significant role in regulating appetite and metabolism, have emerged as potential therapeutic targets for obesity. To better understand the complex relationship between obesity and the gastrointestinal system, we need to delve into the cellular components of the gastrointestinal tract.

The gastrointestinal tract enables food digestion and nutrient absorption, and includes the oral cavity, esophagus, stomach, small intestine, and large intestine^4^. Secretions from various organs, such as the salivary glands, liver, gallbladder, and pancreas, also aid in the digestive process^5^. The luminal side of the gastrointestinal tract is lined by several different types of cells, including EECs, which play an important role as hormone-secreting cells.

The mucosal epithelium of the gastrointestinal tract is in direct contact with the nutrients to be absorbed. The luminal contents are sensed by EECs, which are sparsely distributed in the epithelium along the entire length of the small intestine^6^. EECs constitute less than one percent of the total intestinal epithelial cells, but they are highly heterogeneous within themselves and form the largest specialized endocrine network in the human body. They respond to environmental and nutrient stimuli and provide hormonal control of metabolic homeostasis, regulating various physiological processes such as appetite, intestinal motility, food digestion, and immune response to pathogens^7^. Among the hormones secreted by a subpopulation of EECs is glucagon-like peptide-1 (GLP-1), which has shown promise in the treatment of diabetes and obesity.

GLP-1 is involved in the mediation of glucose-dependent insulin secretion from pancreatic $\beta$ cells, in the regulation of appetite and satiety, GI motility and nutrient metabolism. GLP-1 receptor agonists have been successfully used as a pharmacological approach to the treatment of obesity by reducing appetite and promoting weight loss^8^. Recently, secretin (SCT), another hormone belonging to the glucagon-like peptide family, has emerged as a potential therapeutic target for obesity.

SCT has been shown to induce meal-associated thermogenesis that mediates satiation and thus meal termination via the gut-brown adipose tissue (BAT)-brain axis. Circulating SCT, released from EECs in response to food intake, activates brown fat thermogenesis upon binding to its receptors. Activated BAT thereby exerts a relay function, transmitting the information of nutritional status to the brain, which in turn induces meal termination^9^. A potential therapeutic exploitation of this SCT-mediated signaling axis for control of energy homeostasis presupposes profound understanding of the origin and dynamics of SCT expression and secretion by EECs.

The identification and characterization of a subclass of SCT producing EECs in humans, however, remains challenging. In particular, murine data over the past 15 years have called into question the existence of a definable subclass of uniquely SCT-producing cells, commonly referred to as S cells. In fact, the long-accepted one cell-one hormone dogma seems to be outdated as several studies have shown that different hormones can be produced by a single EEC subtype^10–14^.

The study by Habib et al. (2012) explored the gene expression profiles of EECs in mice, using microarray analysis and qRT-PCR. It revealed significant hormonal overlap among L cells, K cells, and others in the upper small intestine, suggesting these cell types may represent points along a hormonal spectrum rather than distinct entities^10^. Similarly, Fothergill et al. (2017) investigated hormone co-storage and coexpression in EECs of male mice, focusing on the duodenum. Using immunohistochemistry alongside high-resolution microscopy, the research quantified the coexpression of hormones such as serotonin, chromogranin A, secretin, cholecystokinin, ghrelin, and GLP-1 at both cellular and subcellular levels^15^.

Understanding the complexity of EEC sublineages has traditionally been constrained by conventional methods of cell classification. These techniques, including histological analysis, immunohistochemistry, and flow cytometry, provide valuable information but often lack the resolution to fully capture the heterogeneity within rare cell populations. As a result, our knowledge of EEC diversity and function has been limited.

However, breakthroughs in single-cell technology have made it possible to study individual cells at the transcriptional level. These advances have allowed for increasingly fast and accurate analyses, providing new insights into the dynamic nature of the transcriptome that were previously elusive with other technologies^16^. While single-cell technology has led to discoveries in many fields, its applications in the study of enteroendocrine hormones still remain sporadic. Some single-cell transcriptome analyses of sorted EECs have provided further evidence that most EECs contain multiple hormone transcripts and that a specifically SCT-positive cell cluster cannot be defined^12,14, 17^.

Haber et al. conducted a comprehensive single-cell analysis of mouse small intestinal epithelial cells, highlighting the diversity among EECs. They identified and characterized 12 distinct EEC clusters, revealing a surprising heterogeneity beyond the traditionally recognized EEC subtypes. This study showed that in mice key hormones were expressed across multiple EEC subsets, indicating significant crossover among them^12^.

Using a *NEUROG3*-dependent bifluorescent reporter, Gehart and colleagues^14^ profiled the single-cell transcriptomes of murine intestinal EECs along an absolute time axis. They could identify well-separated clusters of mature EECs according to ‘classical marker’ hormone expression but only a minority of these cells dedicated their hormone-encoding RNA exclusively to its respective marker hormone, further supporting hormone coexpression. Interestingly, SCT-positive cells did not form a distinct population, but were present within many other mature cell clusters. The authors propose a temporal plasticity of hormone expression with changing hormone repertoires along cell maturation. In this context, they (and others) hint towards SCT expression especially in mature EECs located towards villi rather than crypts^14^.

The large majority of single-cell EEC analyses was performed on cells of murine origin^12,14^, while investigations into human EECs are scarce^17,18^.

In this context, Beumer et al. already provided valuable insights into human EEC hormone production. They observed that also single organoid-derived human EECs can produce multiple hormones, indicating that in human, similar to murine EECs the one-cell one-hormone dogma does not remain valid. To further substantiate these observations, the study sought to confirm these organoid-derived results through examination of primary human samples. Examining a dataset of 11,302 cells derived from the small intestines of both healthy and diseased individuals, the research successfully identified mRNA profiles for 39 primary human EECs^17^. The collection of such a limited number of primary human EECs by Beumer et al. highlights the challenges posed by the scarcity and diversity of these cells, necessitating extensive sequencing of numerous gut cells to effectively isolate these elusive cells.

However, data on human SCT-expressing cells remain scarce, and the study of human EECs faces unique challenges compared to murine studies, notably the absence of transgenic reporters that facilitate the enrichment of EECs or specific hormone-producing sublineages. Human research often relies on intestinal organoid cultures, where EEC formation can be induced by factors like *NEUROG3* expression, as noted by Beumer et al.^17^ and Sinagoga et al.^19^. Given the potential limitations of these in vitro models, including incomplete differentiation and the influence of *NEUROG3* expression duration on EEC subtype development, there’s a significant interest in augmenting this body of work with analyses of primary human EECs.

In light of these developments, we employ the Gut Cell Atlas (GCA), the most comprehensive single-cell dataset of primary human intestinal cells currently accessible, to explore the intricacies of human enteroendocrine subpopulations. The investigation aims to characterize the hormone repertoires of human EECs and to evaluate the “one EEC-multiple hormones” concept within primary human EECs. Additionally, it seeks to examine *SCT* expression patterns to ascertain the presence of a distinct SCT-positive cell cluster. By providing an extensive overview of the EEC subpopulation and hormone expression profiles, this research endeavors to uncover precise therapeutic targets for obesity, thereby potentially benefiting the health outcomes of millions worldwide.

## Results and discussion

Data quality metrics for the GCA dataset, particularly enteroendocrine cell subpopulations, were carefully evaluated. Quality control involved removing samples with extreme total gene counts, high mitochondrial gene expression, or those identified as doublets. Quality covariates were plotted to set appropriate thresholds for the number of expressed genes and total counts. High mitochondrial gene expression can often signal a high number of apoptotic or lysing cells, which might compromise data quality. Mitochondrial gene expression was investigated in samples organized by region, subregion, diagnosis, and age group. A 20% threshold for mitochondrial gene expression was ultimately applied, ensuring the retention of most EEC cells. Once the quality of the data was assured, the analysis moved on to examine the dynamics of the co-expression of hormones in the EECs, with a particular focus on *SCT* expression.

### Exploratory analysis of SCT expression

S cells have been widely reported in literature as being scattered along the entire length of the small intestine, and the GCA encompasses tissue samples from all the different subregions of the small intestine, as well as from the large intestine and the rectum. The highest frequency is usually expected in the proximal intestine (duodenum) and decreases towards the distal intestine (ileum). According to the “one cell - one hormone” dogma, each EEC subpopulation produces only one hormone specific for that cell type and can be used to label the respective subpopulation. Although the GCA does not include samples explicitly labelled as S cells, this does not necessarily imply an absence of SCT-producing cells in the dataset. The fact that no S cells are labelled in the GCA already suggests that there are no clearly distinguishable, exclusively high SCT-producing cells. To learn more about SCT-producing cells, we examined *SCT* expression in the dataset.

**Figure 1:**
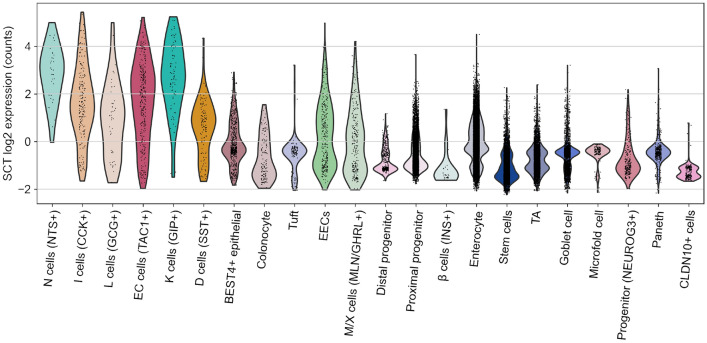
Logarithmic expression of *SCT* counts. The *SCT* read counts (grouped by annotations) after normalization, log2 transformation and batch correction. Many annotations showed moderate *SCT* expression, but no annotation exhibited a significantly elevated *SCT* expression compared to other cell populations. N cells showed the highest expression of *SCT*, followed closely by K cells. EC, I, L, and D cells also showed moderate *SCT* expression.

After performing normalization, log2 transformation, and batch correction of the data, violin plots were used to visualize the expression of each EEC hormone in all annotations present in the dataset (Fig 1, Figs. S1, S2). When comparing the plots, it was noticeable that the expression of *SCT* was very different from the others. For example, unlike *NTS*, it showed many annotations with moderate *SCT* expression, but no annotation with very high *SCT* expression compared to the others. In addition, the plots suggested a possible overlap of hormones produced by different EEC subpopulations.

**Figure 2:**
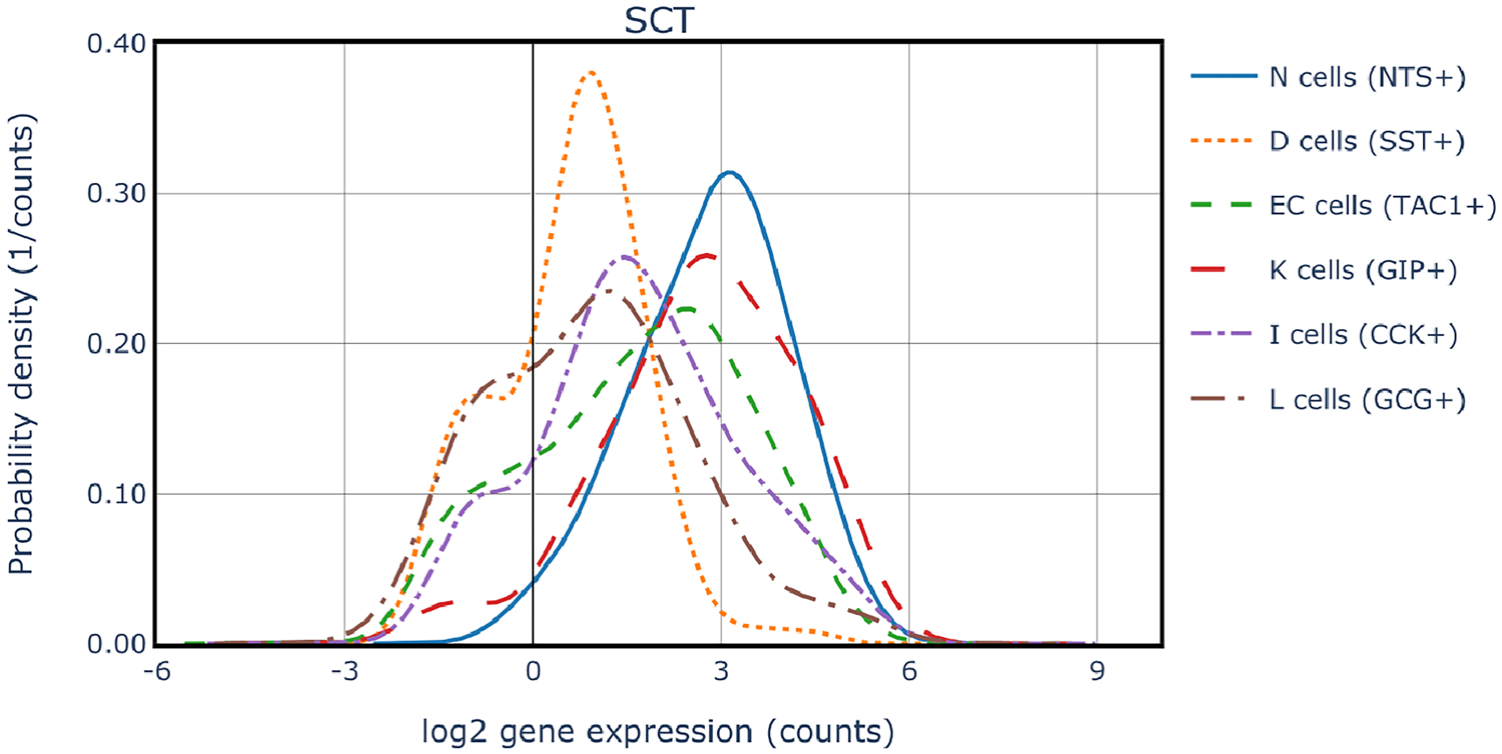
*SCT* normalized read counts in EECs. Several annotations show moderate *SCT* normalized read counts, but there is no annotation with significantly higher *SCT* expression than the others. N cells had the highest *SCT* expression, followed closely by K cells. EC, I, L, and D cells also expressed *SCT*. Additionally, the overlap of hormonal secretion between EECs is particularly striking when *SCT* is examined.

Density plots were used to further investigate the expression distribution of *SCT*, *NTS*, *CCK*, *GIP*, *GCG*, *SST*, and *TAC1* in all EEC subpopulations. *SCT*, *CCK*, *GCG* and other EEC hormones were indeed expressed by several EEC subpopulations (Fig 2, Fig. S3).

The cells expressing *GIP* were K and I cells. *CCK* was expressed by I, K, L, and N cells. *GCG* was expressed by N, L, K and I cells. The cells expressing *NTS* were N and I. *SST* was expressed by D and K cells. *TAC1* was expressed by EC and N cells.

The overlap of gut hormone expression between EECs was particularly striking when their *SCT* expression values were examined. Although N and K cells had the highest *SCT* expression, it was expressed at nearly the same level by most of the other EEC subpopulations. A quantitative approach was taken to describe hormone expression, enabling more precise conclusions.

For *SCT*, *NTS*, *GIP*, *GCG*, *SST*, *CCK*, and *TAC1*, the mean log2 expression value in each annotation was calculated and then sorted in descending order of expression (Fig. S4). Low expression of *NTS* was detected in I and L cells, but as expected, N cells had significantly higher expression than all other annotations. *GIP* was most highly expressed in K cells. It is also expressed in I cells, but to a lesser extent than in K cells. Of all the annotations, *CCK* had the highest expression in I cells, followed by moderate expression in K cells and low expression in L and N cells. *SST* was highly expressed in D cells and moderately expressed in K cells. *GCG* was strongly expressed in N, L and K cells, whereas I cells showed moderate expression of this hormone. Strong expression of *TAC1* was detected in N cells and EC cells, while the other cells did not show *TAC1* expression.

When compared to the expression of *GIP*, *NTS* or *SST*, there is no annotation in the dataset that has a significantly higher mean *SCT* expression than the other annotations. N cells showed the highest expression of *SCT*, closely followed by K cells. Moderate *SCT* expression was also shown by EC, I, L and D cells.

**Figure 3:**
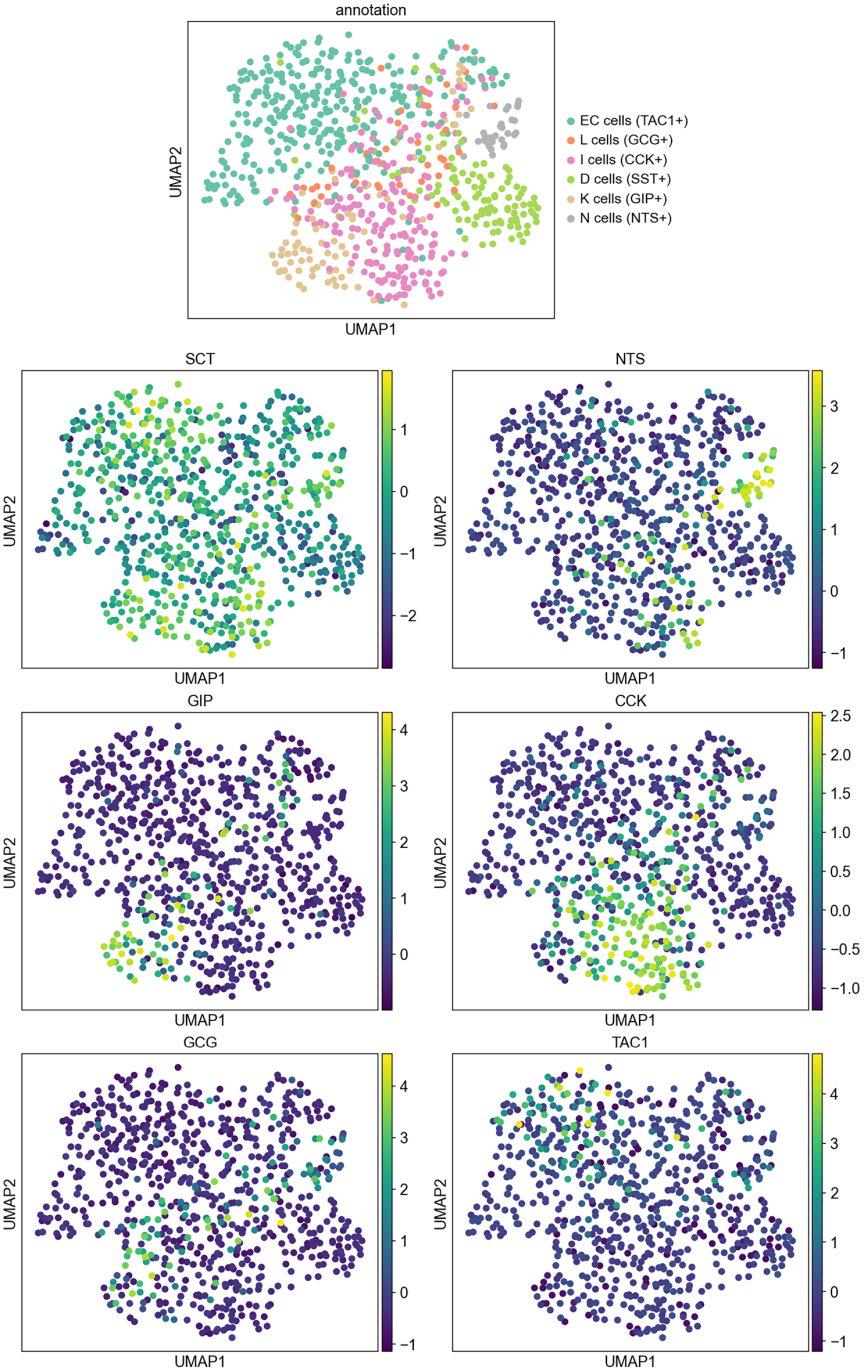
UMAP of EECs with their respective marker hormones colored. The normalized read counts levels of the EEC subpopulations were coloured using their respective marker hormones. While *CCK*, *GIP*, *NTS*, *GCG*, and *TAC1* were mainly expressed in small regions that could be narrowed down, *SCT* showed broad global expression and wasn’t restricted to any particular subpopulation of EECs.

The UMAP (Uniform Manifold Approximation and Projection) of *SCT* expression in EECs provided a better understanding of the *SCT* expression topology (Fig. 3). All EECs where included when generating the UMAP. It was noticeable that the expression of most of the EEC hormones were restricted to their respective EEC subpopulations. *SCT* differs from the other EEC hormones in that it shows a broad expression in all EECs.

The localization of SCT-producing cells along the proximo-distal axis of the gastrointestinal tract revealed a predominance of higher *SCT* expression in the small intestine over the large intestine and rectum (Fig. S5). This pattern aligns with the physiological roles of SCT and the expected distribution of SCT-producing cells, primarily in the proximal part of the gastrointestinal tract^20^.

### SCT dynamics in mature vs. progenitor EECs

**Figure 4:**
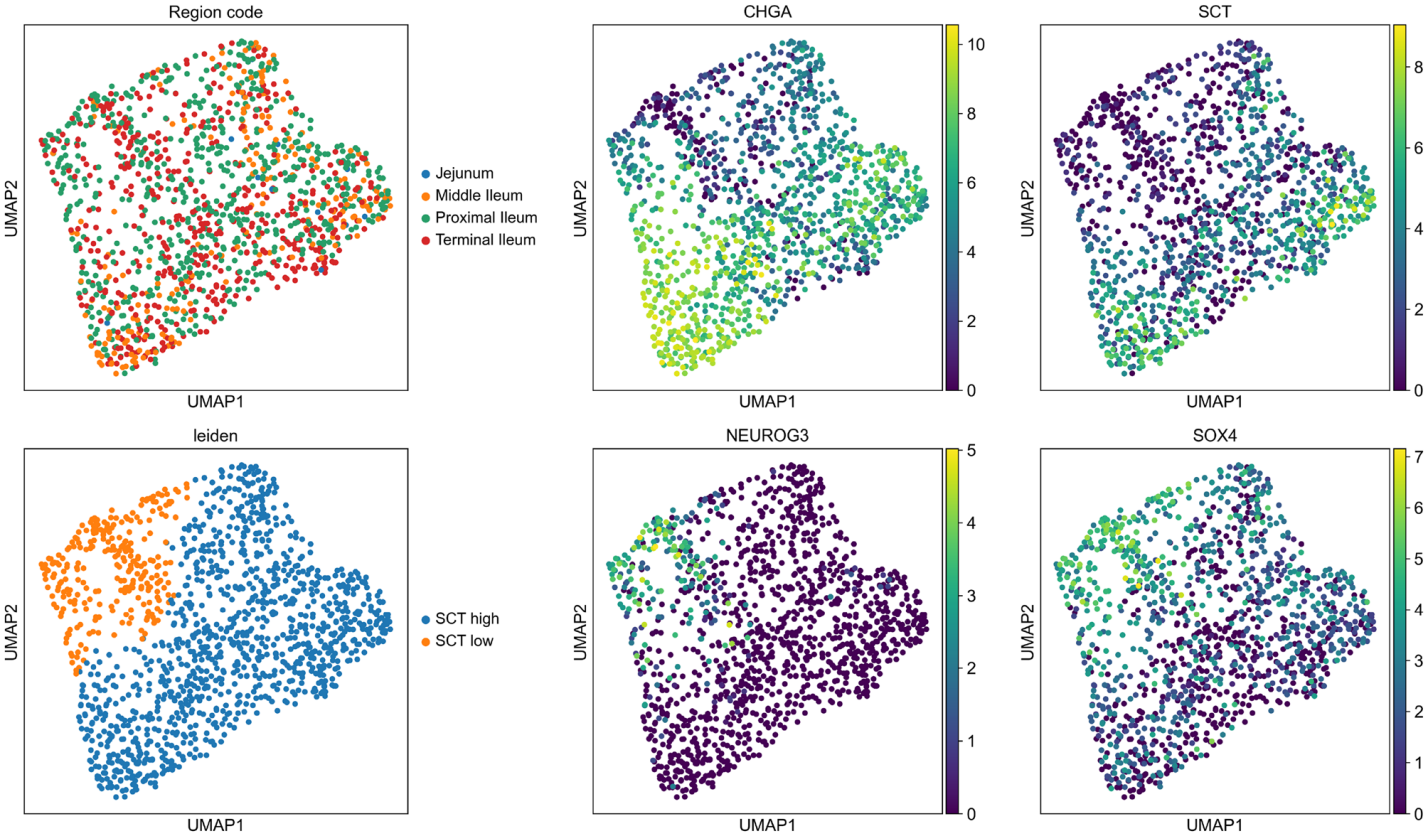
UMAP of *SCT* expression in mature and progenitor EECs. Top-left: Cell distribution by intestinal region with Jejunum (blue), Middle Ileum (orange), Proximal Ileum (green), Terminal Ileum (red). Top-middle: *CHGA* gene expression levels across cells, color-coded from low (purple) to high (yellow). Top-right: *SCT* gene expression with a similar color gradient. Bottom-left: Leiden clustering of cells based on *SCT* expression, distinguishing low *SCT* (orange) as progenitor EECs and high *SCT* (blue) as mature EECs. Bottom-middle and bottom-right: *NEUROG3* and *SOX4* gene expression, respectively.

To ascertain whether cells producing secretin are indicative of mature EECs, we initially identified EEC progenitor cells utilizing the markers Neurogenin 3 (*NEUROG3*) and SRY-Box transcription factor 4 (*SOX4*), as described by Haber et al.^12^ and Beumer et al.^17^. Our findings corroborate that secretin expression peaks in mature EECs. In contrast, progenitor EECs exhibit markedly lower expression levels (Fig 4, Fig. S6). By categorizing cells into subsets based on secretin expression levels, low *SCT* for progenitors and high *SCT* for mature EECs, we then performed differential expression analysis to scrutinize differences between these subsets. *PCSK1N*, *TTR*, *CHGA*, *CHGB* and *SCG5* were upregulated in mature EECs compared to progenitor EECs (Fig. S7). Proprotein convertase subtilisin/kexin type 1 inhibitor (*PCSK1N*) is responsible for the encoding of several bioactive peptides, including proSAAS. This peptide plays a crucial role in negatively regulating *PCSK1* activity and was expressed broadly across all mature EEC populations^21^. Additionally, Transthyretin (*TTR*), known primarily for its role in the transport of thyroid hormone and retinol^22^, may also influence EEC functionality indirectly. The presence of *TTR* in EECs aligns with findings reported by Beumer et al. 2020, suggesting a complex metabolic interplay that could impact secretin dynamics^17^. Furthermore, Chromogranin A (*CHGA*), Chromogranin B (*CHGB*) and Secretogranin V (*SCG5*) are key members of the granin protein family^23^. These proteins are crucial in the secretory processes of EECs, facilitating the storage and release of SCT and other hormones.

### Hormonal co-expression

**Figure 5:**
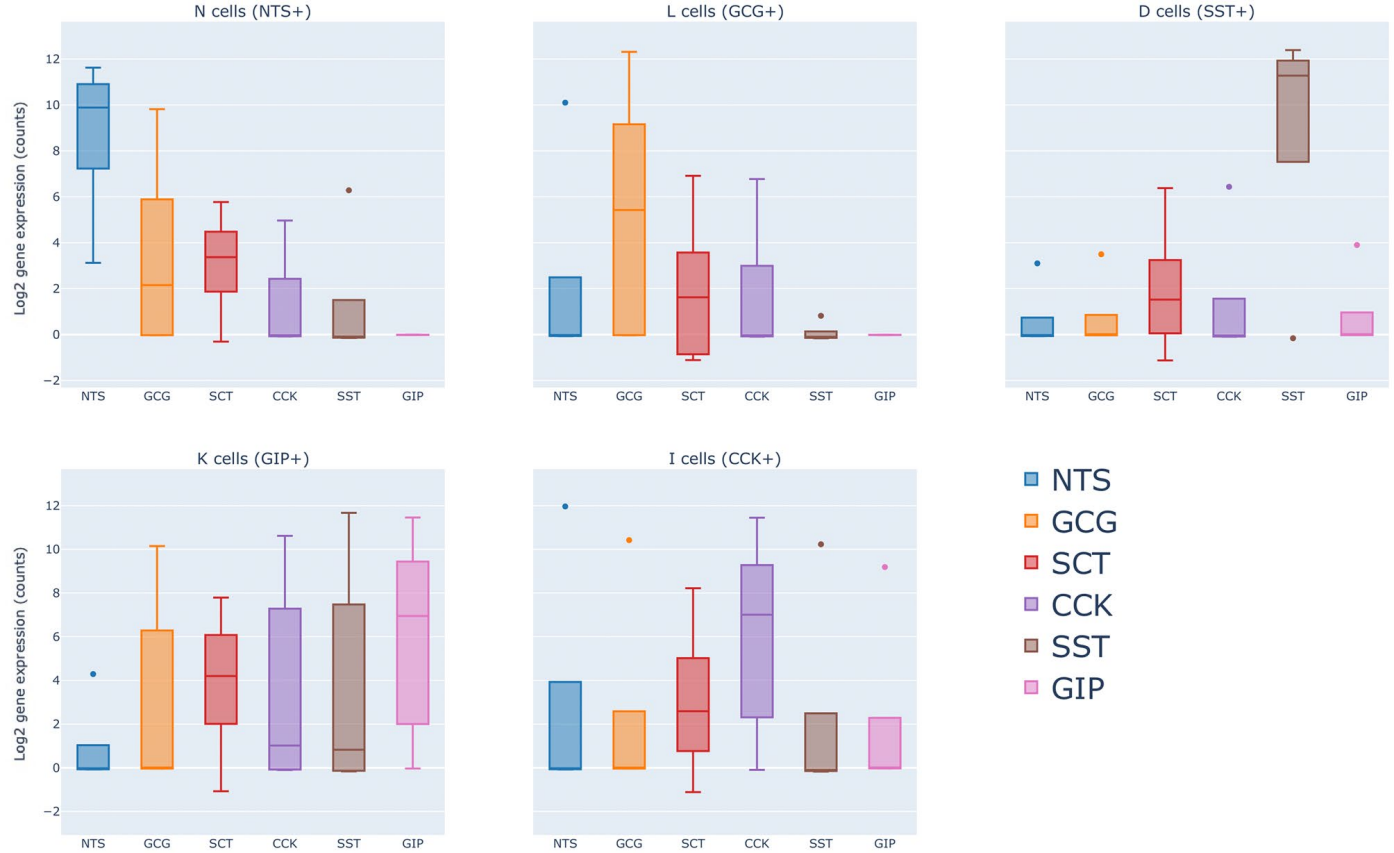
Co-expression of multiple hormones in EECs. *NTS*, *GIP*, *SST*, *CCK*, and *GCG* showed the highest normalized read counts in N, K, D, I, and L cells, respectively. N cells expressed *NTS*, *GCG*, and *SCT*. D cells expressed almost exclusively *SST*, but also *SCT* moderately. K cells expressed *GIP*, followed by *SCT*. *CCK* and *SST* were also expressed in K cells, but the expression was very low. I cells showed a high expression of *CCK* and a moderate *SCT* expression. L cells showed high expression of *GCG*, moderate expression of *SCT*, and low expression of *CCK*. *NTS*, *GIP*, *SST*, *CCK*, and *GCG* showed the highest expression in N, K, D, I, and L cells, respectively.

The normalized read counts distribution of all EEC hormones (SCT, CCK, GIP, NTS, GCG, TAC1) in each of the subpopulations K, I, L, N, D, and EC was examined (Fig 5, Figs. S8, S9).

N cells exhibited expression of *NTS*, *SCT*, and *GCG* in varying levels. D cells, on the other hand, expressed *SST* almost exclusively, while also moderately expressing *SCT*. K cells predominantly expressed *GIP*, with *SCT* following closely behind in expression levels. *CCK* and *SST* were additionally expressed in K cells, although the levels of expression were considerably low. I cells demonstrated a notably high expression of *CCK*, accompanied by a moderate level of *SCT* expression. L cells exhibited a strong expression of *GCG*, a moderate expression of *SCT*, and a comparatively low expression of *CCK*. In summary, *NTS*, *GIP*, *SST*, *CCK*, and *GCG* displayed the highest expression levels in N, K, D, I, and L cells, respectively, indicating distinct hormone profiles for each cell type.

This analysis highlights the diversity in hormone expression among EEC cell types, showing distinct hormone profiles for each, yet revealing significant multi-hormone co-expression within individual cells. It broadens the “one cell-multiple hormone” concept, initially grounded in murine research, to include primary human tissue, highlighting the multifaceted nature of EEC functionality in humans.

## Conclusion

The growing prevalence of obesity has spurred interest in hormonal therapies targeting EEC hormones, which are crucial in regulating appetite and energy balance. As obesity is frequently linked to the dysregulation of these hormones, treatments focusing on these pathways could prove effective in managing obesity and associated metabolic disorders.

Investigating the expression of EEC hormones in EEC cells has been challenging due to their scarcity and heterogeneity, rendering bulk RNA sequencing insufficient. Nevertheless, the emergence of single-cell RNA sequencing and large-scale single-cell sequencing projects now enables the isolation and analysis of individual EEC cells’ gene expression, offering a deeper understanding of their function and possible therapeutic targets.

While numerous studies have highlighted the importance of EECs and the hormones they secrete, the bulk of this research has primarily focused on primary murine, murine organoid, or human organoid tissues, with investigations using scRNA-seq on primary human tissue to study EEC cells being scarce. The Gut Cell Atlas emerged as a significant resource in this context, offering the most extensive scRNA-seq dataset of primary human intestinal cells to date. Our analysis using this comprehensive dataset not only consolidates the broad *SCT* expression found in murine, murine organoid, and human organoid EECs but also offers a more precise quantification of hormone expression by primary human EECs.

Specific analyses into SCT-expressing cells reject the presence of any cell population that exhibits significantly elevated *SCT* expression compared to other cell populations, previously referred to as S cells. Our investigation reveals that SCT production is, in fact, a collective effort among various EEC subpopulations. This finding not only validates observations from studies on murine EECs but extends them to human EECs, underscoring a fundamental similarity across species.

In addition to these insights, our research provides compelling evidence that *SCT* expression is predominantly observed in mature EECs. In stark contrast, progenitor EECs display significantly lower levels of *SCT* expression. This supports the notion that *SCT* expression is a hallmark of EEC maturation. Further analysis into the differential expression between these subsets revealed notable upregulation of *PCSK1N*, *TTR*, *CHGB*, and *SCG5* in mature EECs, implicating these genes in the secretory capabilities and hormonal regulation of mature EECs.

As single-cell sequencing becomes increasingly cost-effective, defining EEC subpopulations based on hormone co-expression could potentially offer a more effective alternative for the future categorization of EEC cells. An emerging field of interest is spatial transcriptomics, which could potentially identify if SCT-producing cells form clusters within the tissue. This could provide further insight into the functionality and organization of these cells.

While our study provides valuable insights into EEC hormones, it is crucial to acknowledge its limitations. Although single-cell sequencing offers significant insights into the complexity of EEC cells, we recognize that it may not be equally effective for all cell types due to differences in cell shape and size. This introduces the possibility that we may have overlooked selectively SCT-expressing cells in our investigation. We mainly focused on mRNA expression patterns, which do not always reflect protein levels. Therefore, our findings should be validated at the protein level to confirm these expression patterns.

Overall, the results of this investigation emphasize the importance of future research into the secretion and regulation of SCT and other EEC hormones. Understanding the complex interactions between these hormones and their effects on digestion and other physiological processes could have significant implications for the development of treatments for digestive disorders and other related conditions.

## Materials and methods

### Gut cell atlas

The Gut Cell Atlas (GCA) is part of the Human Cell Atlas effort to collect and categorize all cells found in the human body. Approximately 430,000 cells from different gastrointestinal tract regions of healthy fetal, infant, and adult donors are part of the GCA. One of the project’s main goals is to map cell lineages and study intra- and intercellular variability in the intestinal tract. The large number of intestinal cells provided by the Gut Cell Atlas is essential for identifying very rare cell populations. However, it is important to note that enteroendocrine cells, despite their crucial roles, are remarkably scarce. Among the vast number of cells catalogued, only 830 enteroendocrine cells were identified, underscoring their rarity.

The GCA sampled tissue from deceased transplant organ donors which was then processed and analysed using a combination of cell isolation, magnetic-activated cell sorting (MACS) enrichment, droplet-based scRNA-seq, and other techniques. Subclusters of enteroendocrine cells were identified based on known genes expressed, including M/X cells (*MLN*/*GHRL*), D cells (*SST*), $\beta$ cells (INS), L cells (*GCG*), N cells (*NTS*), K cells (*GIP*), I cells (*CCK*) and enterochromaffin cells (*TPH1*). More details on methodology and results can be found in their publication^24^.

### Quality control

The Gut Cell Atlas included only those samples in its dataset that met some initial quality criteria. Samples with less than 500 genes and more than 50% expressed mitochondrial genes were removed. Also removed were genes expressed in fewer than four samples. Then Scrublet, a tool for identifying doublets in single-cell RNA-seq data, was used to exclude doublets with a cut-off value of 0.25. This initial filtering was only a first step in ensuring high data quality and needed to be complemented by more stringent filters.

Plotting the number of genes by counts, the total counts per sample, and the proportion of expressed mitochondrial genes provided a better understanding of the distribution of these quality covariates across samples (Fig. S10). The samples in the data set contained between 500 and 8000 genes. The number of total counts per sample ranged from 400 to approximately 120,000.

Furthermore, the proportion of mitochondrial genes expressed in samples grouped by region, subregion, diagnosis, and age group was examined (Fig. S11). All regions showed an overall high expression of mitochondrial genes, with samples from the appendix, rectum, and lymph nodes showing the highest expression. The small intestine, where the EEC samples are located, and the colon were less affected by this phenomenon. When the samples were grouped by age, it was evident that the fetal samples had the lowest expression of mitochondrial genes while the adult samples had the highest expression. This was also evident when the samples were grouped according to subregions. Fetal samples had moderate expression of mitochondrial genes, whereas adult samples had very high expression. This may be due to the fact that the adult tissues were obtained postmortem from deceased organ donors and these cells may have already been apoptotic. An increased expression of mitochondrial genes was also noticeable when grouping intestinal samples by sample annotation. While Goblet, Paneth, Tuft, Microfold, Colonocyte and Enterochromaffin (EC) samples were most affected ($\ge$ 15–20%), EEC samples (L, I, D, K, N, and EC) showed a lower expression ($\approx$ 10%) of mitochondrial genes than the above annotations (Fig. S12).

Current publications recommend a 5-10% threshold for the proportion of mitochondrial genes to remove damaged or low-quality samples. However, the validity of using such a uniform threshold for different species, single-cell technologies, tissues, and cell types has not been adequately evaluated. A systematic analysis of human tissues found a higher average proportion of mitochondrial genes in human tissues than in mouse tissues^25^.

The effect of different thresholds for quality metrics on the number of total and EEC samples in the dataset must always be considered, so the relationship between the selected threshold for the proportion of mitochondrial genes and the number of samples below that threshold was examined (Fig. S13). Of the total 68,000 intestinal epithelial samples, 830 were EEC. Using the widely accepted threshold of 10%, only 32,000 (48%) of the total samples would have met this requirement, but nearly 72% (600) of the EEC samples would have remained in the dataset.

Based on the information obtained from the plotted quality metrics, an upper limit of 5500 and 40,000 was set for the number of expressed genes and the number of total counts, respectively. A permissive threshold of 20% was used to filter the proportion of mitochondrial genes expressed. Samples above the set thresholds were filtered out of the dataset. This retained almost all (92%) of the valuable EEC samples and 62% (42,000) of the total samples.

The three covariates of data quality, i.e. the proportion of mitochondrial genes, the number of genes by counts, and the number of total counts expressed, were plotted again. The defined thresholds for the covariates were verified to be effective, resulting in only the desired samples being kept in the dataset (Fig. S14).

Additionally, the gene expression profiles of each EEC subpopulation were analyzed using two Scanpy functions, sc.pl.highest_expr_genes and sc.pl.violin. The sc.pl.highest_expr_genes function computes the mean fraction of counts assigned to each gene within a cell and returns the top n genes with the highest mean fraction across all cells. The sc.pl.violin function generates violin plots that show the distribution of gene expression across different cell types, with the widest and highest peak representing the cell type with the highest expression of the examined gene/hormone.

The expected marker gene for each EEC subpopulation was the highest expressing gene in both sc.pl.highest_expr_genes and sc.pl.violin. This was true for all EEC subpopulations except for EC cells, which had the highest *TAC1* expression in sc.pl.violin but did not list *TAC1* as one of the top genes in sc.pl.highest_expr_genes (Fig. S15). *MLN*, another EEC hormone often attributed to EC cells, was highly expressed by the EC subpopulation. EC cells showed high expression of *SCT*, which is the focus of this investigation. Therefore, EC cells were retained in the dataset for further analysis.

### List of analysis tools

- Python (3.9.7)
- NumPy (1.22)
- Pandas (1.4.2)
- Matplotlib (3.5.2)
- Scanpy (1.9.1)
- AnnData (0.9)
- UMAP (0.5)
- BBKNN (1.5.1)

### Supplementary Information

Supplementary Information 1.

Supplementary Information 2.

Supplementary Information 3.

Supplementary Information 4.

Supplementary Information 5.

Supplementary Information 6.

Supplementary Information 7.

Supplementary Information 8.

## Data Availability

The single-cell dataset used for this study were made available from the Gut Cell Atlas and are accessible through the following link: https://cellgeni.cog.sanger.ac.uk/gutcellatlas/epi_raw_counts02.h5ad. Raw sequencing data are available at ArrayExpress with accession numbers E-MTAB-9543, E-MTAB-9536, E-MTAB-9532, E-MTAB-9533, and E-MTAB-10386.

## Code availability

The code generated during this study is available at Github: https://github.com/technodoc/secretin_project.git.
