# Supplementary material for: Single-cell transcriptome analysis reveals secretin as a hallmark of human enteroendocrine cell maturation
Source: Sci Rep. 2024 Jun 12;14:13525. doi: 10.1038/s41598-024-63699-0 (PMC11169271; doi:10.1038/s41598-024-63699-0)
Supplement: Supplementary file 1 — Supplementary Information 1. [file 41598_2024_63699_MOESM1_ESM.pdf]

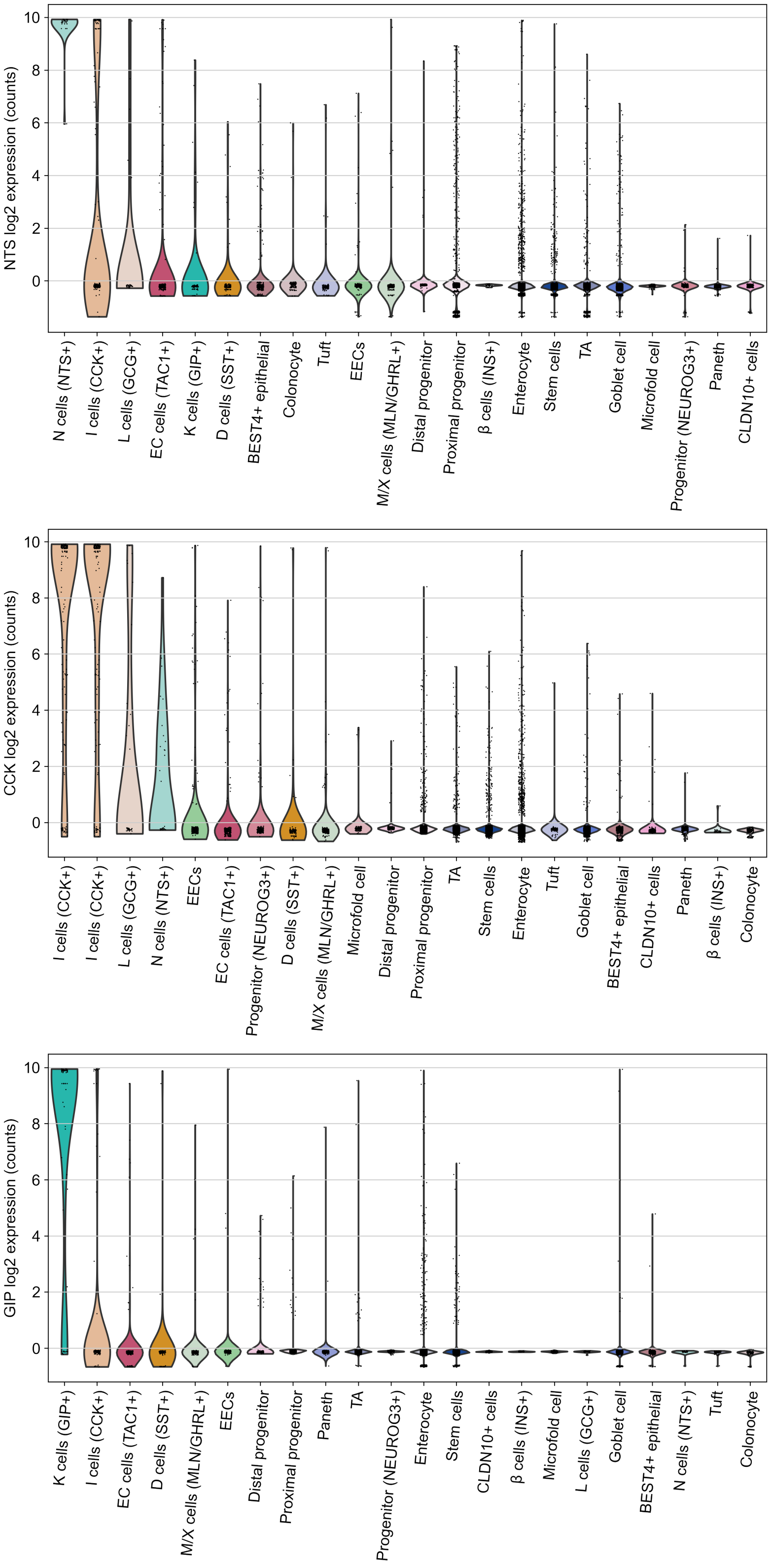

Fig. S1. Logarithmic expression of *NTS*, *CCK*, and *GIP* counts sorted by annotation. *NTS*, *CCK*, and *GIP* normalized read counts (grouped by annotation) after normalization, log2 transformation and batch correction. *NTS*, *CCK*, and *GIP* showed the highest expression in N, I, and K cells, respectively. An overlap in expression of EEC hormones is noticeable.

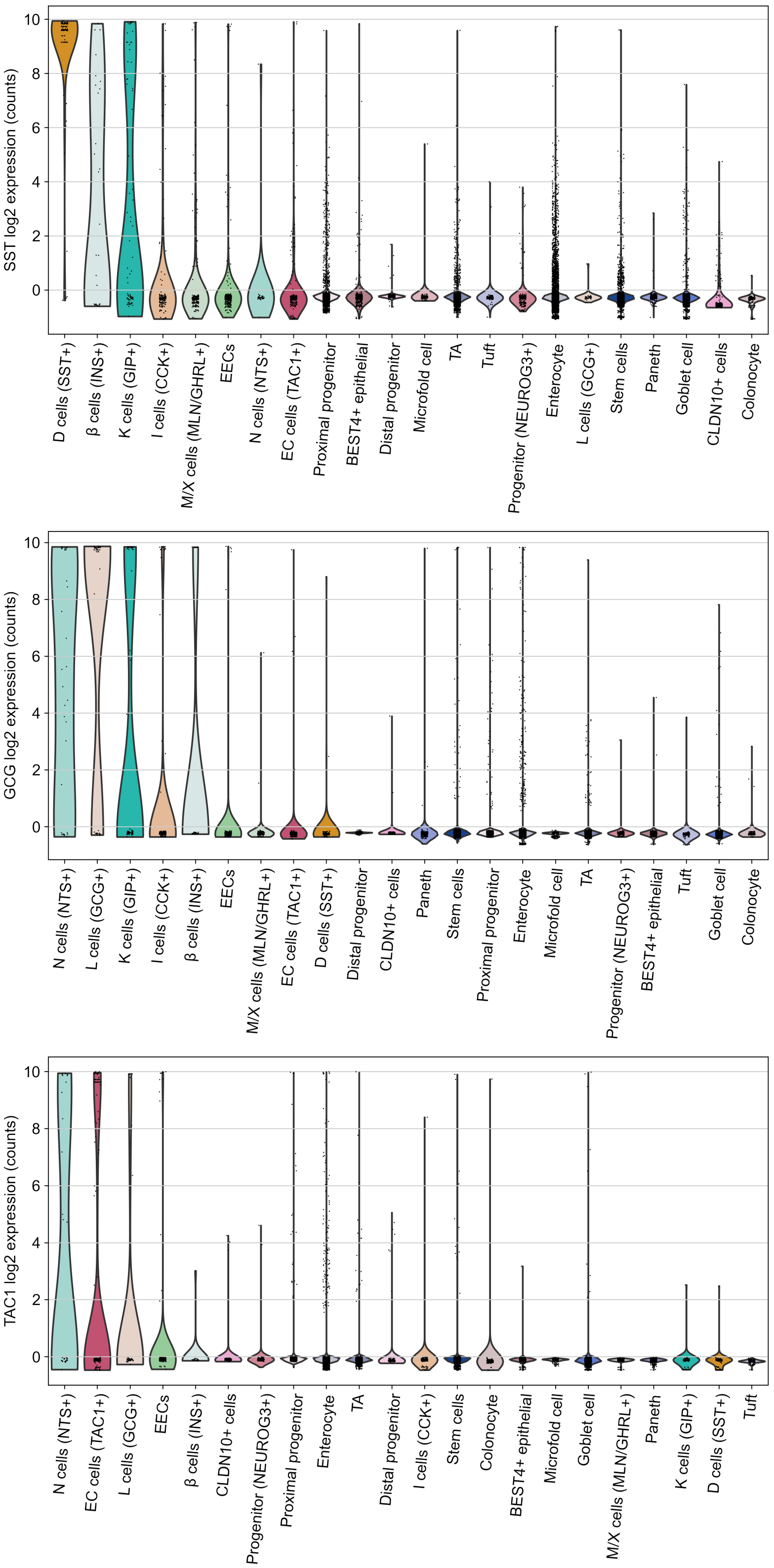

Fig. S2. Logarithmic expression of *SST*, *GCG*, and *TAC1* counts sorted by annotation. *SST*, *GCG*, and *TAC1* read counts (grouped by annotation) after normalization, log transformation and batch correction. *SST*, *GCG*, and *TAC1* showed the highest expression in D, N, and EC cells, respectively. An overlap in expression of EEC hormones is noticeable.

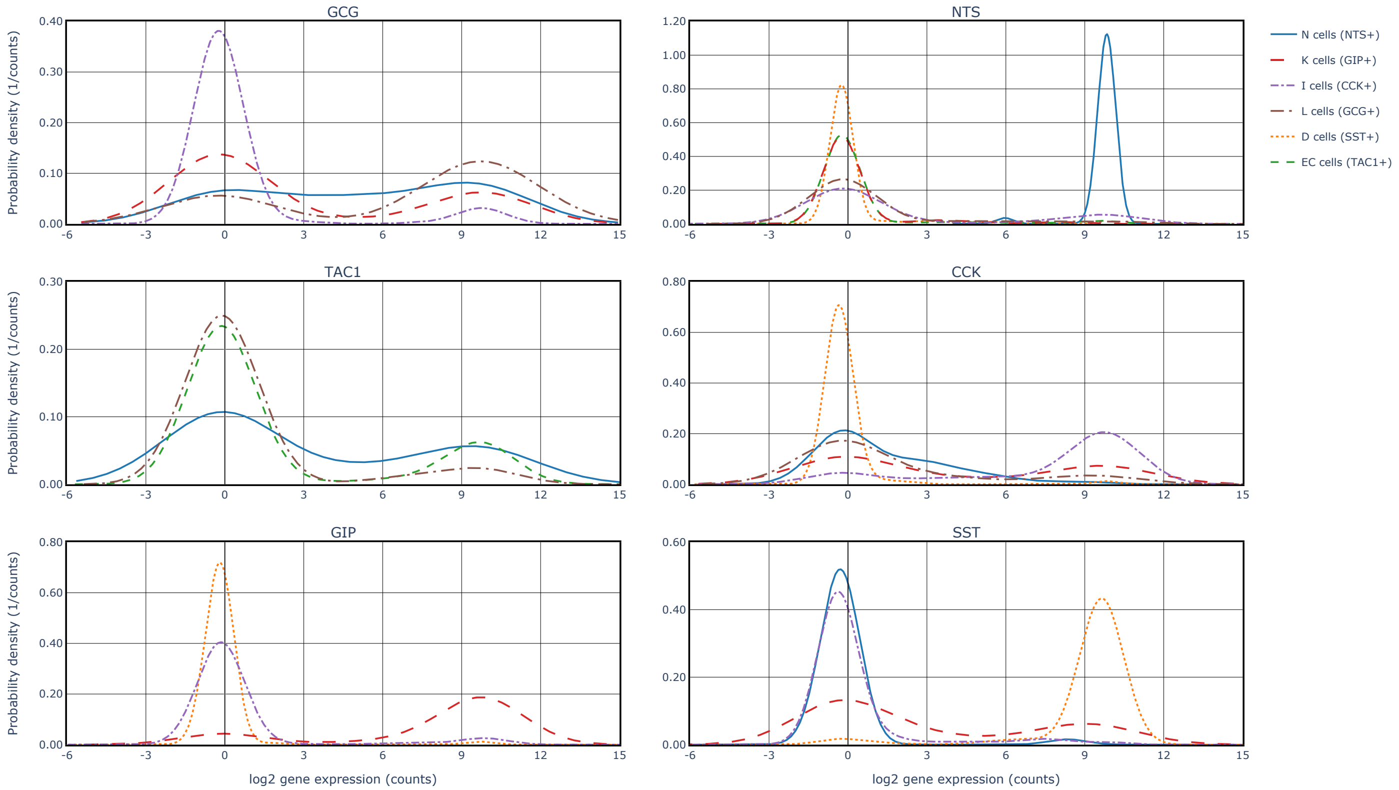

Fig. S3. Expression of EEC hormones by different EECs. EECs are interesting, not only because of their ability to express *SCT*, *CCK*, *GCG*, and other EEC hormones that are functionally very different, but also because of the overlap in hormone secretion between EEC subpopulations. For example, *SCT* is highly expressed by N and K cells but also by almost all other EEC subpopulations, which is quite striking. In addition, EEC hormones were expressed as follows: *GIP*: K and D cells; *CCK*: I, K, L, and N cells; *GCG*: N, L, K, and I cells; *NTS*: N and I cells; *SST*: D and K cells; *TAC1*: EC and N cells. In this probability density plot, the genes with a mean log2 normalized read counts of 0 and a probability density greater than 1 have been removed for better visibility. These lines did not provide meaningful information for the analysis and their removal allows for a clearer representation of the remaining data.

Annotation	Mean SCT count expression
N cells (NTS+)	+2.80742
K cells (GIP+)	+2.65536
EC cells (TAC1+)	+1.65510
I cells (CCK+)	+1.64597
L cells (GCG+)	+0.92631
D cells (SST+)	+0.57719

Annotation	Mean GIP count expression
K cells (GIP+)	+7.74337
I cells (CCK+)	+0.61093
EC cells (TAC1+)	-0.01786
D cells (SST+)	-0.02287
L cells (GCG+)	-0.12543
N cells (NTS+)	-0.14598

Annotation	Mean TAC1 count expression
N cells (NTS+)	+3.39755
EC cells (TAC1+)	+2.09717
L cells (GCG+)	+0.94697
I cells (CCK+)	-0.08703
K cells (GIP+)	-0.13501
D cells (SST+)	-0.15212

Annotation	Mean SST count expression
D cells (SST+)	+9.06742
K cells (GIP+)	+3.00430
I cells (CCK+)	+0.29734
N cells (NTS+)	-0.05204
EC cells (TAC1+)	-0.07705
L cells (GCG+)	-0.25477

Annotation	Mean GCG count expression
N cells (NTS+)	+7.64171
L cells (GCG+)	+6.18063
K cells (GIP+)	+4.54275
I cells (CCK+)	+2.74183
EC cells (TAC1+)	-0.05604
D cells (SST+)	-0.08345

Annotation	Mean NTS count expression
N cells (NTS+)	+9.71588
I cells (CCK+)	+2.04028
L cells (GCG+)	+0.84272
EC cells (TAC1+)	+0.38872
K cells (GIP+)	+0.23838
D cells (SST+)	+0.04877

Annotation	Mean CCK count expression
I cells (CCK+)	+7.45393
K cells (GIP+)	+3.85512
L cells (GCG+)	+1.74040
N cells (NTS+)	+1.39054
EC cells (TAC1+)	-0.00428
D cells (SST+)	-0.10746

Fig. S4. Mean expression levels of EEC hormones in each annotation. After normalization, log2 transformation, and batch correction of the data, the mean *SCT*, *GIP*, *TAC1*, *SST*, *GCG*, *NTS*, and *CCK* normalized read counts are calculated for all EEC populations. The results show an overlap in the hormonal expression of EECs. *SCT* is expressed nearly at the same level in N, K, I, and L cells. *SST*, *GIP*, *NTS*, and *CCK* showed, as described by the "one cell one hormone" dogma, the highest expression in D, K, N, and I cells, respectively. *GCG* showed the highest expression in N cells, followed closely by L cells. *TAC1* was expressed the most by N and EC cells.

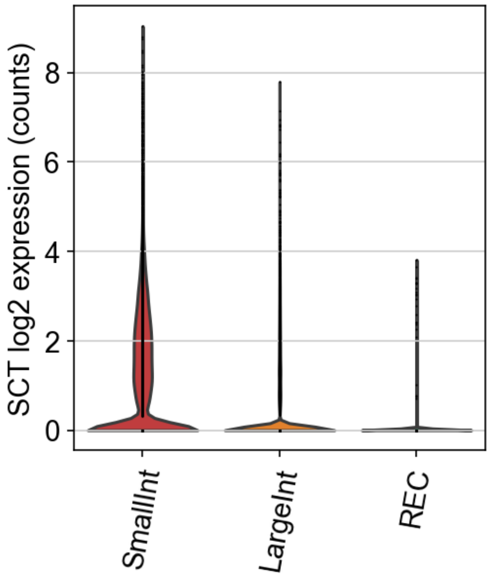

Fig. S5. Distribution of *SCT* Expression in the Gastrointestinal Tract. Violin plot showing *SCT* expression across the gastrointestinal tract, with regions labeled as Small Intestine (SmallInt), Large Intestine (LargeInt), and Rectum (REC). The plot illustrates the log2-transformed expression counts of secretin-producing cells, indicating a higher expression of *SCT* in the small intestine compared to the large intestine and rectum.

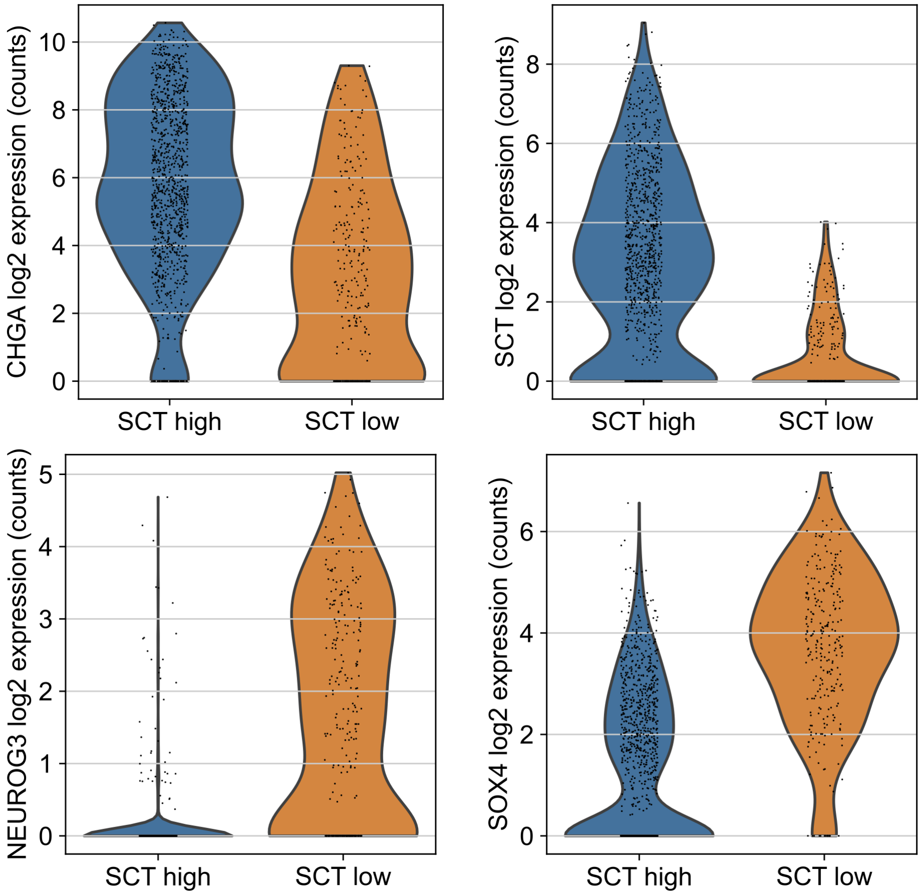

Fig. S6. Secretin Dynamics in Mature vs. Progenitor EECs. Violin plots depicting the expression levels of *CHGA* and *SCT*, and progenitor markers *NEUROG3* and *SOX4* in *SCT* high and *SCT* low enteroendocrine cells (EECs). The plots on the left compare the expression of *CHGA* (top) and *NEUROG3* (bottom) in cells with high versus low levels of *SCT*. The plots on the right display the expression of *SCT* (top) and *SOX4* (bottom) under the same conditions. Each plot shows the distribution and density of gene expression counts, with dots representing individual cells. Higher density regions of the plot are wider, illustrating the concentration of cells with similar expression levels. These visualizations indicate higher *CHGA* and *SCT* expressions in mature EECs (*SCT* high), whereas *NEUROG3* and *SOX4* are more expressed in progenitor EECs (*SCT* low).

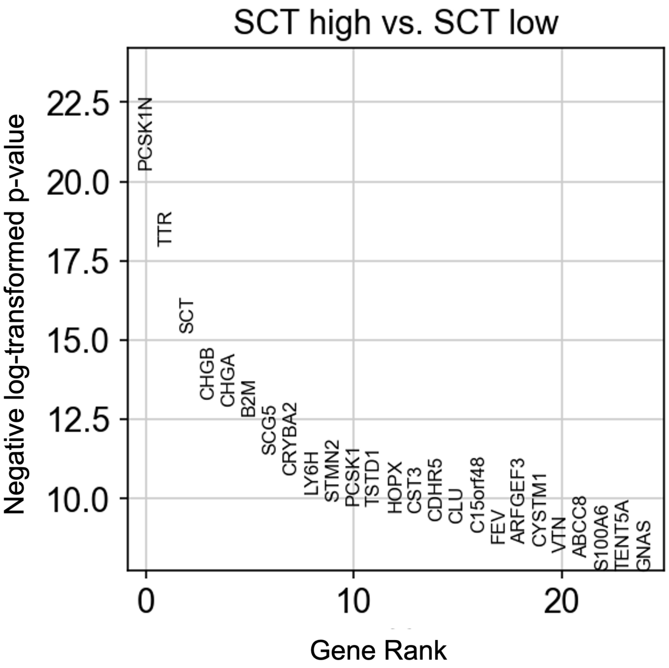

Fig. S7. Differential gene expression analysis between *SCT* high and *SCT* low enteroendocrine cells (EECs). The plot displays gene names ranked by their negative log-transformed p-values, indicating the level of differential expression. Genes such as *PCSK1N*, *TTR*, *SCT*, *CHGB* and *CHGA* are among the most significantly upregulated in *SCT* high EECs. The x-axis represents the gene rank based on the significance of expression difference, and the y-axis represents the negative log-transformed p-values, highlighting the statistical significance of each gene's differential expression.

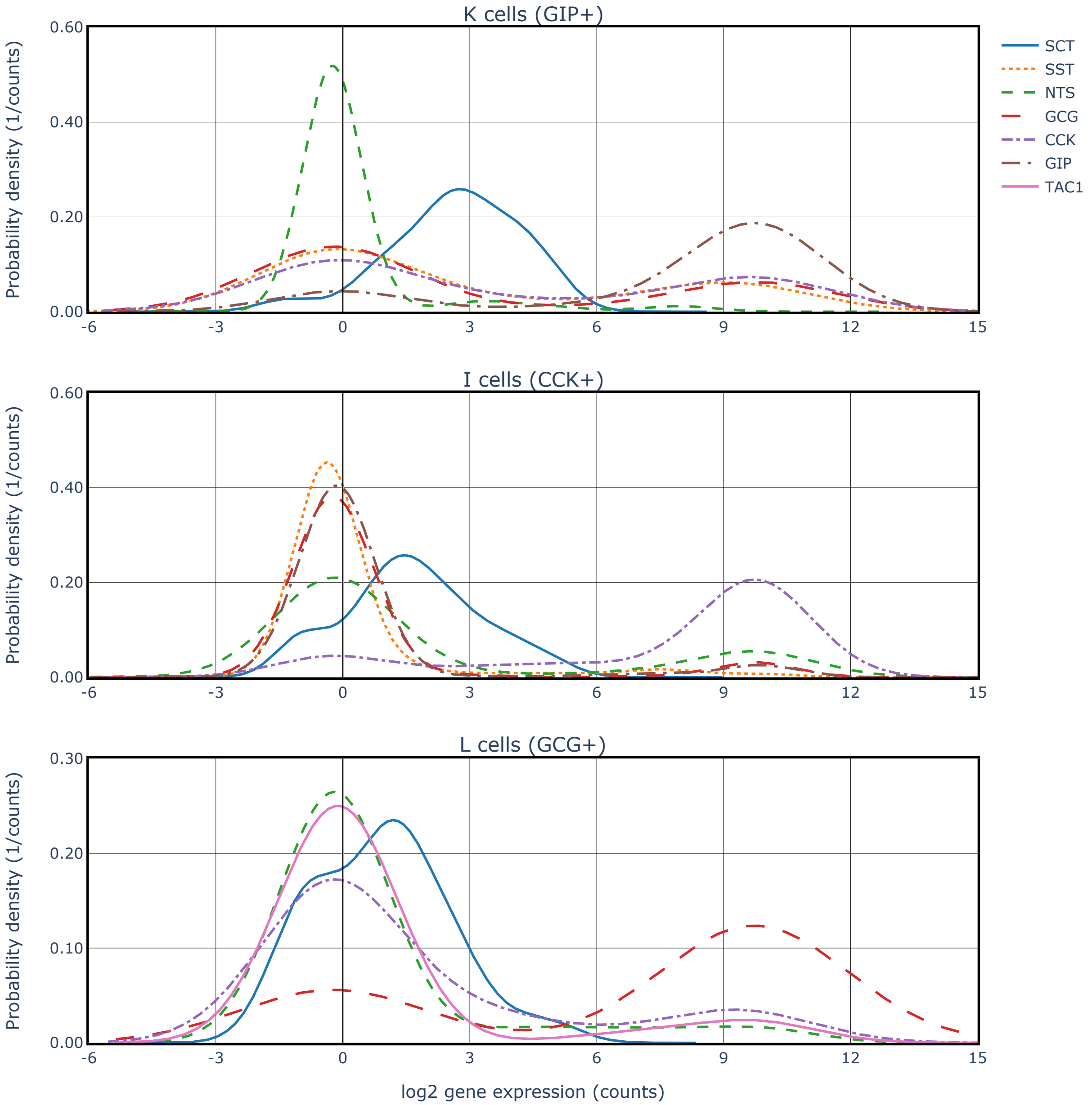

Fig. S8. Expression of EEC hormones in K, I, and L cells. Expression of EEC hormone counts in K, I, and L cells. Multiple EEC hormones are expressed in all three subpopulations. *GIP*, *CCK*, and *GCG* show the highest normalized read counts in K, I, and L cells, respectively. All three subpopulations showed a positive *SCT* expression. In this probability density plot, the genes with a mean normalized log2 read counts of 0 and a probability density greater than 1 have been removed for better visibility. These lines did not provide meaningful information for the analysis and their removal allows for a clearer representation of the remaining data.

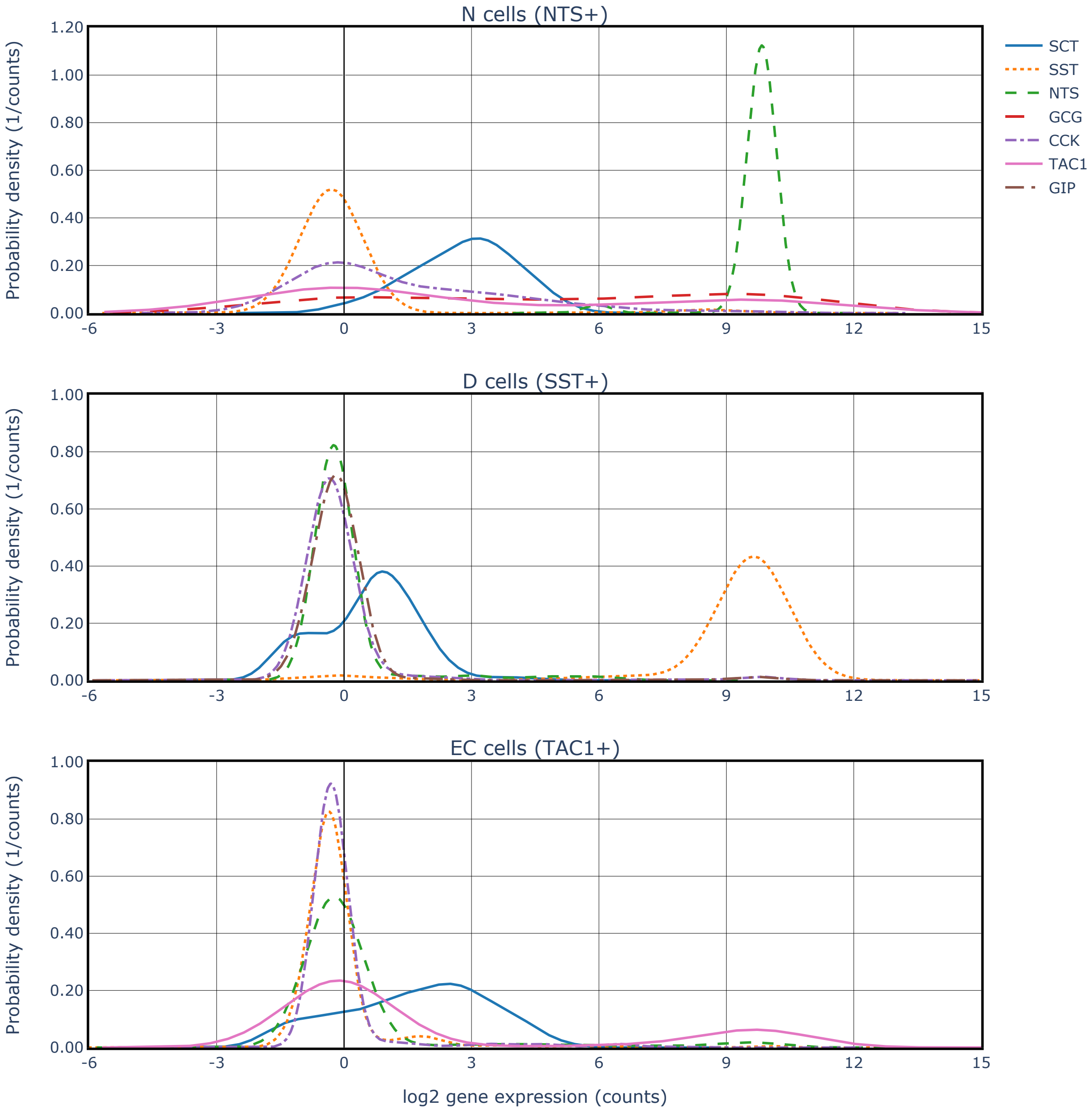

Fig. S9. Expression of EEC hormones in N, D, and EC cells. Expression of EEC hormone counts in N, D, and EC cells. Multiple EEC hormones are expressed in all three subpopulations. *NTS*, *SST*, and *TAC1* show the highest normalized read counts in N, D, and *TAC1* cells, respectively. All three subpopulations showed a positive *SCT* expression. In this probability density plot, the genes with a mean normalized log2 read counts of 0 and a probability density greater than 1 have been removed for better visibility. These lines did not provide meaningful information for the analysis and their removal allows for a clearer representation of the remaining data.

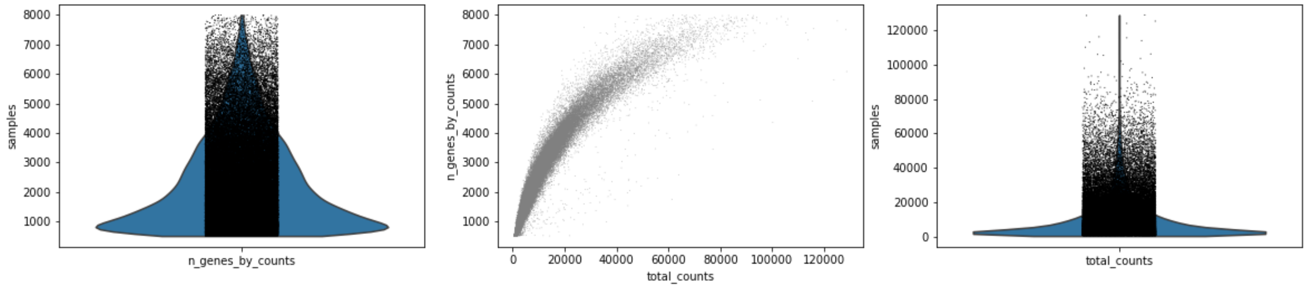

Fig. S10. Quality covariates plotted before filtering. The left plot shows the number of genes expressed in each sample. Each sample expresses between 500 and 8,000 genes. The number of total counts per sample is shown in the right plot, and the values range from 400 to about 120,000 counts for some samples. The number of genes by counts compared to total counts are shown together in the middle plot to better visualize the joint effect they have in filtering.

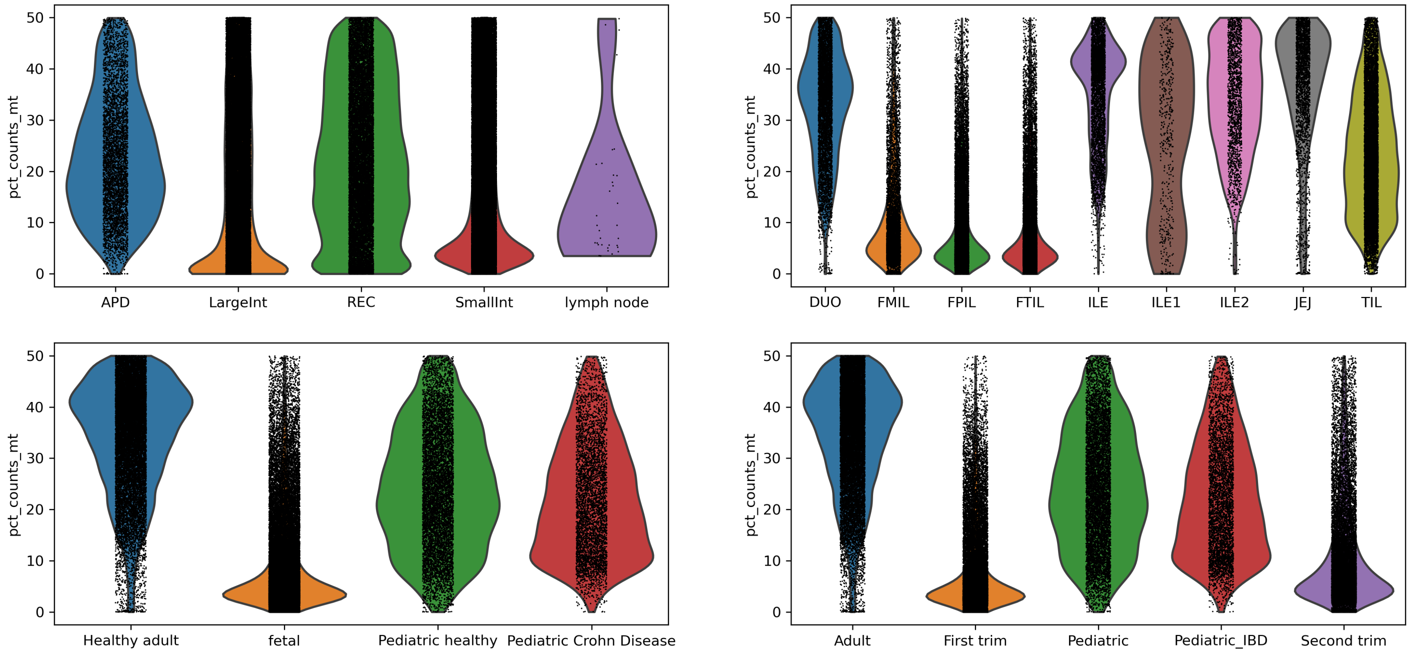

Fig. S11. Mitochondrial genes proportion in the Gut Cell Atlas Dataset. Proportion of mitochondrial genes grouped by region (upper left), subregion (upper right), diagnosis (lower left), and age group (lower right). Adult samples show overall high expression, while fetal samples show low expression of mitochondrial genes.

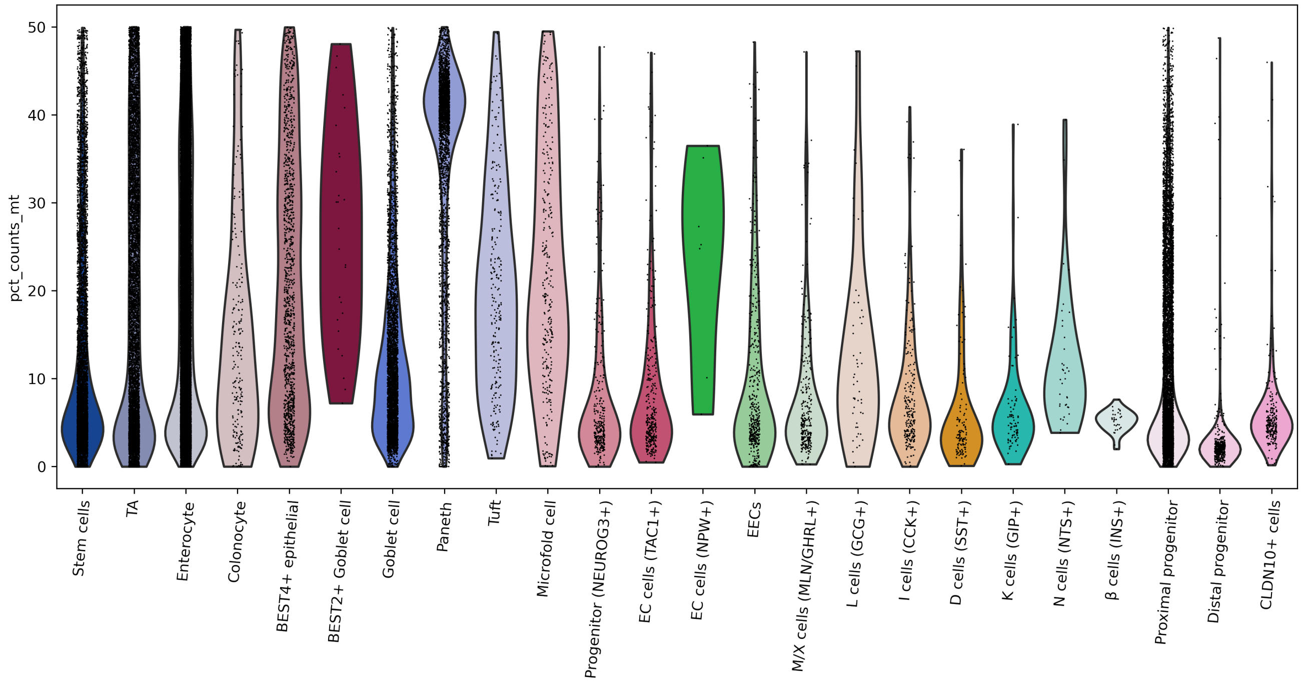

Fig. S12. Proportion of mitochondrial genes grouped by dataset annotations. The annotations present in the dataset express mitochondrial genes in varying proportions. Overall, increased expression is observed. Paneth cells show very high expression of mitochondrial genes. Goblet, tuft, microfold, colonocyte, and enterochromaffin (EC) samples also show relatively high expression ($\geq 15\%$). Enteroendocrine samples (L, I, D, K, N, M, and EC), on the other hand, are not as affected ($\approx 10\%$) by this phenomenon.

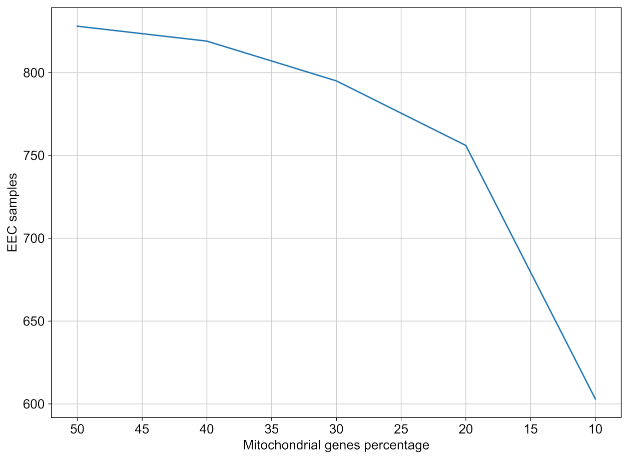

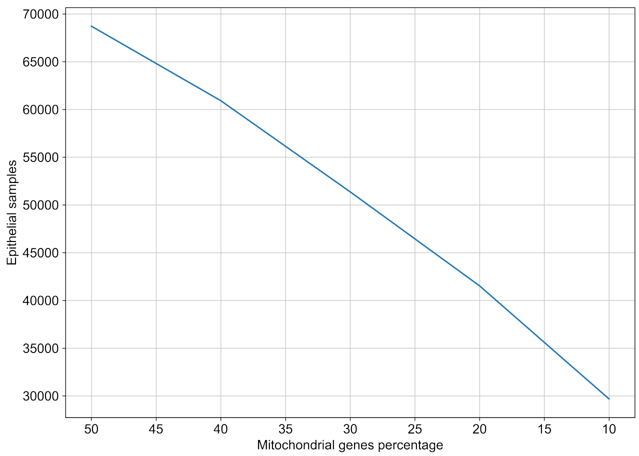

Fig. S13. Samples number dependence on mitochondrial threshold. The left plot shows the number of enteroendocrine cell samples in the dataset for different thresholds of the proportion of mitochondrial genes. The right plot shows the number of total samples in the dataset for different thresholds of the proportion of mitochondrial genes. For very stringent thresholds ($\leq 10\%$), most samples would be filtered out. For less stringent thresholds ($\geq 20\%$), most enteroendocrine cells and total samples are retained.

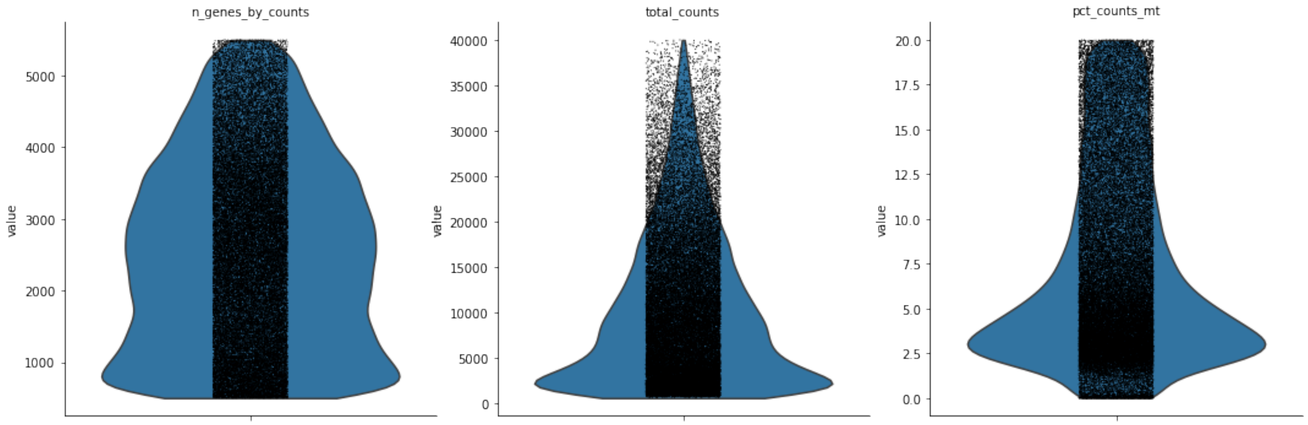

Fig. S14. Quality covariates plotted after filtering. Based on the information obtained from the quality metrics, an upper limit of 5,500 and 40,000 was set for the number of expressed genes and the number of total counts, respectively. A permissive threshold of 20% was used to filter the proportion of mitochondrial genes expressed. Samples above the set thresholds were filtered out of the dataset. The three covariates of data quality, i.e., the proportion of mitochondrial genes, the number of genes by counts, and the number of total counts expressed, were plotted again. The defined thresholds for the covariates were verified to be effective, resulting in only the desired samples being kept in the dataset.

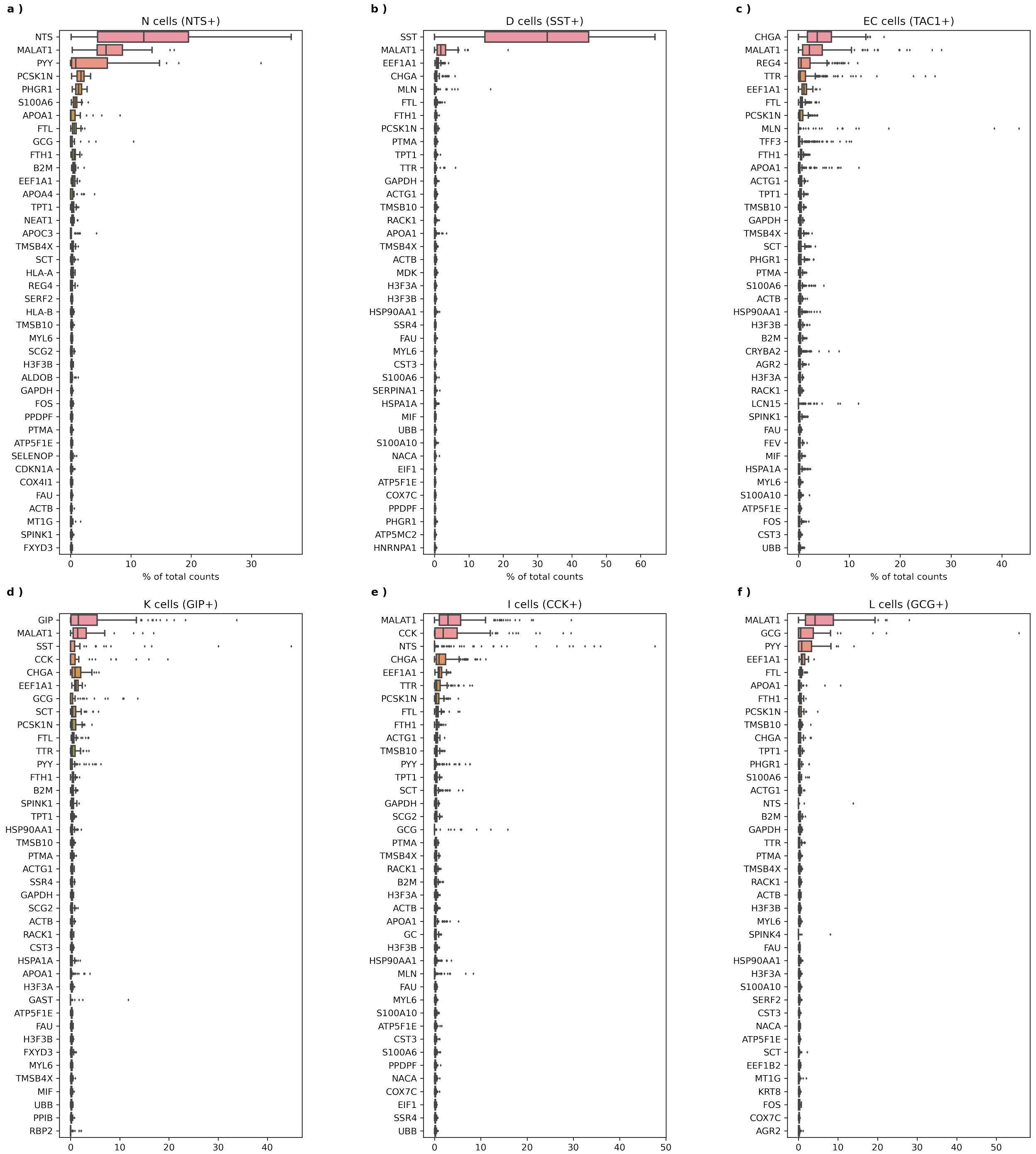

Fig. S15. Highest expressed genes in EEC subpopulations. The figure consists of six subplots labeled a) - f). Each subplot shows a boxplot of the highest expressed genes in a specific type of cell: N, D, EC, K, I, and L cells, respectively. The genes shown in each subplot were identified using the sc.pl.high_expr_genes function (Scanpy), which calculates the fraction of counts assigned to each gene over all cells in the dataset. The boxplots in each subplot show the normalized read counts levels of the 40 top genes with the highest mean fraction over all cells. The genes are sorted by decreasing mean fraction, with the gene with the highest mean fraction at the top of each boxplot. *NTS*, *SST*, *MLN*, *GIP*, *CCK* and *GCG* are the most highly expressed enteroendocrine hormones for N, D, EC, K, I and L cells respectively.
