# Supplementary material for: Single-cell transcriptome analysis reveals secretin as a hallmark of human enteroendocrine cell maturation
Source: Sci Rep. 2024 Jun 12;14:13525. doi: 10.1038/s41598-024-63699-0 (PMC11169271; doi:10.1038/s41598-024-63699-0)
Supplement: Supplementary file 8 — Supplementary Information 8. [file 41598_2024_63699_MOESM8_ESM.docx]

Supporting information

S1 File. I cell samples. H5ad file with I cell samples that passed quality control. Quality control involved removing samples with extreme total gene counts, high mitochondrial gene expression, or those identified as doublets.

S2 File. K cell samples. H5ad file with K cell samples that passed quality control. Quality control involved removing samples with extreme total gene counts, high mitochondrial gene expression, or those identified as doublets.

S3 File. L cell samples. H5ad file with L cell samples that passed quality control. Quality control involved removing samples with extreme total gene counts, high mitochondrial gene expression, or those identified as doublets.

S4 File. N cell samples. H5ad file with N cell samples that passed quality control. Quality control involved removing samples with extreme total gene counts, high mitochondrial gene expression, or those identified as doublets.

S5 File. D cell samples. H5ad file with D cell samples that passed quality control. Quality control involved removing samples with extreme total gene counts, high mitochondrial gene expression, or those identified as doublets.

S6 File. EC cell samples. H5ad file with EC cell samples that passed quality control. Quality control involved removing samples with extreme total gene counts, high mitochondrial gene expression, or those identified as doublets.
